# Effects of human arylamine *N*-acetyltransferase I knockdown in triple-negative breast cancer cell lines

**DOI:** 10.1002/cam4.415

**Published:** 2015-01-28

**Authors:** Jacky M Tiang, Neville J Butcher, Rodney F Minchin

**Affiliations:** School of Biomedical Sciences, University of QueenslandSt Lucia, Queensland, 4072, Australia

**Keywords:** Filopodia, invasion, MDA-MB-231, metastasis, *N*-acetyltransferase

## Abstract

Expression of human arylamine *N*-acetyltransferase I (NAT1) has been associated with various cancer subtypes and inhibition of this enzyme with small molecule inhibitors or siRNA affects cell growth and survival. Here, we have investigated the role of NAT1 in the invasiveness of breast cancer cells both in vitro and in vivo. We knocked down NAT1 using a lentivirus-based shRNA approach and observed marked changes in cell morphology in the triple-negative breast cancer cell lines MDA-MB-231, MDA-MB-436, and BT-549. Most notable was a reduction in the number and size of the filopodia protrusions on the surface of the cells. The loss of filopodia could be rescued by the reintroduction of NAT1 into the knockdown cells. NAT1 expression was localized to the lamellipodia and extended into the filopodia protrusions. In vitro invasion through Geltrex was significantly inhibited in both the MDA cell lines but not in the BT-549 cells. The expression of Snail increased when NAT1 was knocked down, while other genes associated with mesenchymal to epithelial transition (vimentin, cytokeratin-18, and Twist) did not show any changes. By contrast, both N-cadherin and *β*-catenin were significantly reduced. When MDA-MB-231 cells expressing shRNA were injected in vivo into BALB/c nu/nu nude mice, a significant reduction in the number of colonies that formed in the lungs was observed. Taken together, the results show that NAT1 can alter the invasion and metastatic properties of some triple-negative breast cancer cells but not all. The study suggests that NAT1 may be a novel therapeutic target in a subset of breast cancers.

## Introduction

The arylamine *N*-acetyltransferases (NAT; EC 2.3.1.5) were first discovered as drug-metabolizing enzymes that catalyze the transfer of an acetyl group from acetyl coenzyme A to the nitrogen of arylamine compounds. There are two isozymes expressed in humans (NAT1 and NAT2) that differ in their tissue distribution and substrate specificity 1,2. Because the NATs metabolically activate and detoxify arylamine and heterocyclic amine carcinogens, there has been an extensive interest in their role as risk factors in a range of human cancers 3. Both NAT1 and NAT2 are genetically polymorphic resulting in rapid and slow acetylator phenotypes. Rapid NAT2 acetylators are reportedly at greater risk of colon and breast cancers, while slow acetylators are at greater risk of bladder and pancreatic cancers. Cancer risk and NAT1 phenotype, however, are less clearly defined 4.

The NATs are structurally similar to the family of cysteine proteases that contain a Cys-His-Asp catalytic triad at their active site 5. This triad is conserved in most NATs from bacteria to humans 6. For NAT1, the active-site cysteine undergoes acetylation in the absence of substrate, which regulates the stability of the protein 7. It is sensitive to oxidative stress and can be reversibly and irreversibly inactivated by oxidants such as hydrogen peroxide, peroxynitrite, and S-nitrosothiols 8–10. More recently, NAT1 was shown to be inhibited by a range of small molecule drugs including cisplatin 11, disulfiram 12, and the 5-benzylidenerhodanine derivative Rhod-o-hp 13.

The effect of NAT1 on cell growth and survival was first reported by Adams et al. who observed that normal breast epithelial cells overexpressing NAT1 showed enhanced growth and survival as well as resistance to etoposide, although etoposide is not a substrate for the enzyme 14. Later studies showed that NAT1 is a potential biomarker for breast cancer 15 and higher expression is associated with increased disease mortality and recurrence 16. In addition, NAT1 methylation status is significantly increased in tamoxifen-resistant breast tumors 17. None of these studies have associated the effects of NAT1 on cancer cell survival with the acetylation activity of the enzyme.

Many proteins have been shown to possess multiple, often unrelated, functions in the cell 18 Examples of multitasking enzymes include cytochrome C, which participates in mitochondrial electron transport chain as well as apoptotic pathways 19 and ERK2, a widely studied protein kinase that can also function as a transcription repressor 20. More recently, the protein MRI1 has been shown to modulate RhoA-dependent cell invasion as well as participate in the methionine salvage pathway as an isomerase 21. This multitasking demonstrates the diversity that a single gene product can have in the cell.

In the present study, we have used the invasive triple-negative human breast cancer cells MDA-MB-231, MDA-MB-436, and BT-549 to investigate the effects of shRNA-mediated NAT1 knockdown on cell morphology and on invasive capacity in vitro. We also investigated in vivo lung colony formation following intravenous injection of the MDA-MB-231 cells in mice. In addition to its function as a xenobiotic metabolizing enzyme, the results presented here suggest that NAT1 may also act as a regulator of cancer cell growth and survival.

## Materials and Methods

### Cell lines and cell culture

MDA-MB-231, MDA-MB-436, and BT-549 cells were obtained from the American Type Culture Collection (Manassas, VA) and cultured in Roswell Park Memorial Institue (RPMI) 1640 supplemented with 10% Turbo calf serum (Life Technologies, Melbourne, VIC, Australia) and 1% penicillin/streptomycin, and maintained at 37°C in a humidified atmosphere of 5% CO_2_ in air. Cell lines were authenticated by short tandem repeat profiling (Queensland Institute of Medical Research, Brisbane, QLD, Australia).

### shRNA and generation of lentivirus

S-fold software was used to predict sequences for silencing of human NAT1 22. A scrambled sequence was used as a control. The sequences (Control: GGAATCTCATTCGATGCAT; shNAT1: GGGAACAGTACATTCCAA) were cloned into pLL3.7 Lenti-lox vector containing the reporter gene enhanced green fluorescent protein (EGFP) and the lentivirus was generated as described previously^23^. Briefly, the pLL3.7 and packaging vectors were cotransfected into 293T cells and the resulting supernatant was collected after 36 h. The supernatant was centrifuged at 4°C and virus filtered through a 0.45 *μ*m filter membrane. Prior to transducing cells, virus particles were concentrated with Vivaspin 20 concentrators (Sartorius Stedim Biotech GmbH, Goettingen, Germany) according to the manufacturer's instructions.

### Generation of stable cell lines

Cells (1 × 10^5^) were transduced with 100 *μ*L concentrated virus particles carrying either the scrambled shRNA sequence or NAT1-directed shRNA sequence in the presence of 6 *μ*g/mL polybrene for 20 h. Following transduction, cells were expanded and sorted using a FACSVantage SE DiVa Cell Sorter (BD Biosciences, San Jose, CA) to exclude nontransduced cells.

### Assay of NAT1 activity

Cells were washed with PBS, resuspended in buffer (20 mmol/L Tris [pH 7.4], 1 mmol/L ethylenediaminetetraacetic acid (EDTA), and 1 mmol/L dithiotreitol), and lysed on ice by sonication. Cell lysates were then centrifuged at 4°C and the supernatant assayed for NAT1 activity as described previously 24. Protein concentrations were determined using Bio-Rad protein assay dye (Hercules, CA).

### Western blotting

Cell lysates containing equal amounts of protein were electrophoresed on 12% polyacrylamide gels, transferred to nitrocellulose membranes, and immunoblotted using primary antibodies. Anti-vimentin (VI-01), anti-*β*-catenin (ab32572), anti-Twist (ab5087), anti-E-cadherin (ab1416), and anti-cytokeratin-18 (C-04) antibodies were purchased from Abcam (Cambridge, UK). Anti-N-cadherin (13A9) and anti-tubulin (DM1A) antibodies were obtained from Calbiochem (Darmstadt, Germany). Anti-Snail (3879) was obtained from Cell Signalling Technology (Danvers, MA). Following horseradish peroxidase-conjugated secondary antibody incubation, immunoblots were detected using the Immun-Star™ HRP Chemiluminescent kit (Bio-Rad, Hercules, CA).

### Immunocytochemistry

Cells were seeded at a density of 2.5 × 10^4^/cm^2^ on coverslips. After 48 h, cells were fixed in 4% paraformaldehyde for 20 min at room temperature in CSK buffer (100 mmol/L KCl, 300 mmol/L sucrose, 2 mmol/L ethyleneglycoltetraaceticacid (EGTA), 2 mmol/L MgCl_2_, 10 mmol/L PIPES, pH 7.4) and permeabilized with 0.5% Triton X-100/PBS for 5 min at room temperature. Coverslips were then blocked in 3% BSA/PBS for 1 h at room temperature. Following primary antibody incubation, cells were washed with PBS and incubated with fluorescent-conjugated secondary antibodies (Life Technologies) for 1 h at room temperature. Alexa-Fluor 647-conjugated phalloidin (Life Technologies) was used to stain F-actin. Coverslips were mounted using Vectashield mounting medium with DAPI (Vector Laboratories Inc., Burlingame, CA) and sealed. Slides were examined using an Olympus BX61 confocal microscope (North Ryde, NSW, Australia).

### Chemotaxis and invasion assays

Migration (chemotaxis) assays were performed by seeding 1 × 10^5^ cells onto 8 *μ*m polycarbonate pore membrane inserts (Corning, Lowell, MA) in RPMI 1640 supplemented with 0.2% Turbo calf serum. The inserts were then placed into a 24-well plate containing culture medium supplemented with 10% Turbo calf serum as the chemoattractant. Cells migrating through the pores over 24 h were fixed with methanol for 2 min and then stained with 1% toluidine blue in 1% borax for 2 min. For invasion assays, the procedure was the same as the migration assay except the 8 *μ*m polycarbonate pore membrane inserts that were overlaid with (test) or without (control) 10–15 *μ*g Geltrex (Life Technologies) and the inserts were dried overnight at 37°C prior to cell seeding. In both assays, photographs of three random fields per membrane were taken and the number of cells was counted to calculate the average number of cells per field that had transmigrated. In the invasion assay, the percent cell invasion (% CI) was calculated as:

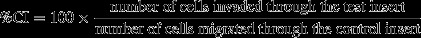


### In vivo metastasis in mice

Lentivirus-transduced MDA-MB-231 cells were trypsinized and washed with PBS. Then 2 × 10^6^ cells in 100 *μ*L PBS were injected into the lateral tail vein of 6–8 week old BALB/c nu/nu nude mice. Mice were sacrificed 56 days after xenografting and lungs were removed. For colony counting, lungs were inflated with 1% low melting agarose and stained with Bouin's solution (Sigma-Aldrich, St Louis, MO) for 24 h. For histology analysis, lungs were perfused with PBS-buffered 4% paraformaldehyde, followed by inflation with 1% low melting agarose. The lungs were then embedded in OCT medium and cryosectioned at 10 *μ*m. Cryosections were counterstained with 4′,6-diamidino-2-phenylindole (DAPI) to reveal nuclei. Animal work was approved by The University of Queensland animal ethics committee.

### Data analysis

Statistics were performed using Prism 5 (GraphPad Software Inc., San Diego, CA) and data expressed as mean ± SEM. Statistical comparisons between two groups were assessed by Student's *t*-tests or one-way analysis of variance (ANOVA) assuming significance at a *P* value of 0.05 or less.

## Results

### NAT1 knockdown alters breast cancer cell morphology and invasion in vitro

**Figure 1:**
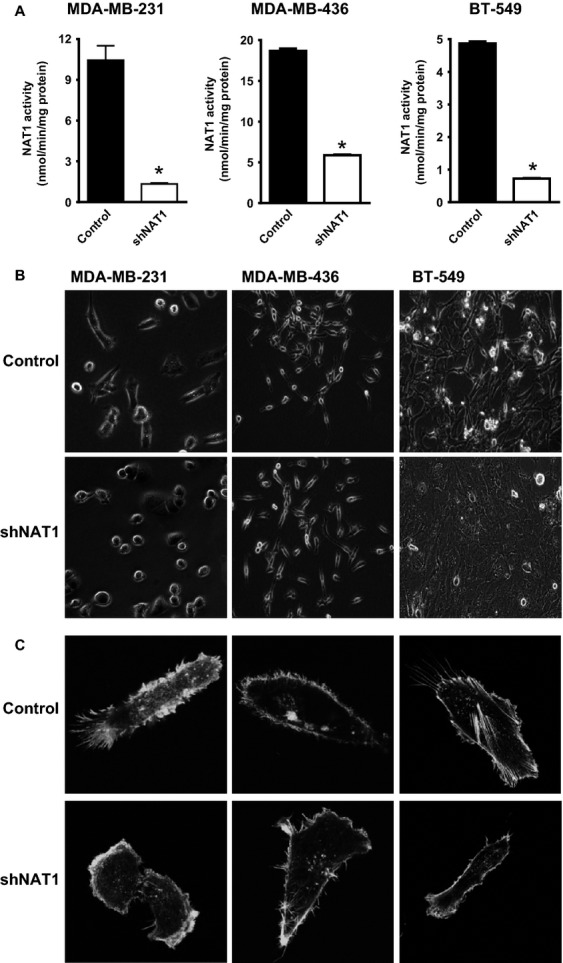
Arylamine *N*-acetyltransferase I (NAT1) knockdown alters breast cancer cell morphology. (A) NAT1 activity in different breast cancer cells following transduction with shNAT1 lentivirus. Results are mean ± SEM, *n* = 3. Asterisk indicates *P *<* *0.05. (B) Change in cell morphology following NAT1 knockdown. (C) Distribution of F-actin in different breast cancer cells following NAT1 knockdown demonstrated by confocal imaging of phalloidin-stained cells.

We first examined the effect of inhibiting NAT1 in breast cancer cell lines. All three lines used are adenocarcinomas that have undergone epithelial to mesenchymal transition (EMT) or exhibit a post-EMT phenotype. Transduction of each cell line with a shRNA lentivirus directed against NAT1 (shNAT1) resulted in a significant reduction in NAT1 activity compared to a scrambled control (Fig.1A). Preliminary studies showed that nontransfected cells and cells with little NAT1 knockdown had a growth advantage that led to their dominance in culture over time. To minimize this, transduced cells were sorted by FACS to select for EGFP-positive cells only. The knockdown in NAT1 activity following sorting varied from 70% in the MDA-MB-436 to 88% in the MDA-MB-231 cells.

Loss of NAT1 led to a change in cell morphology. Figure1B shows that control MDA-MB-231 cells displayed a typical elongated epithelial phenotype but shNAT1-treated cells were more rounded, lacking cell protrusions, and with less spreading on the surface of the culture plate. shNAT1-treated BT-549 cells were more cobblestoned in appearance compared to control cells. For the MDA-MB-436 cells, morphological changes were less obvious at the light microscopy level. However, under confocal microscopy where cellular actin was stained with phalloidin (Fig.1C), all three cell lines demonstrated smaller, collapsed filopodia, and localization of actin to the inner surface of the cell membrane, possibly indicative of a reduction in stress fiber contractility 25.

**Figure 2:**
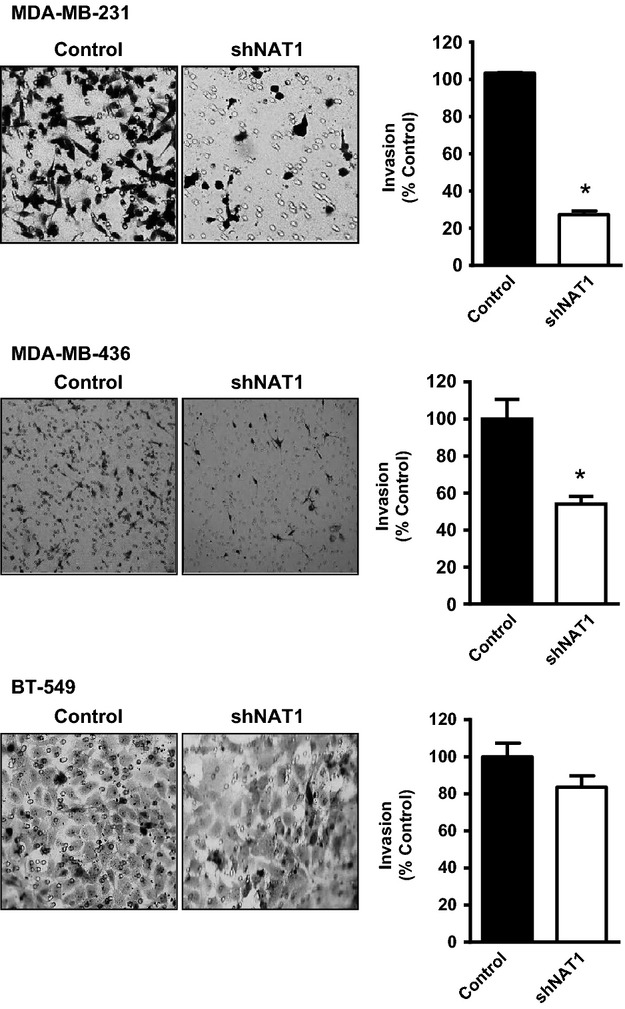
Arylamine *N*-acetyltransferase I (NAT1) knockdown inhibits breast cancer cell invasion in vitro. Control and shNAT1 cells stained with toluidine blue after invasion through Geltrex are shown on the left for the three different breast cell lines. Quantification of the invaded cells is shown on the right. Results are mean ± SEM, *n* = 3. Asterisk indicates *P *<* *0.05.

In vitro invasion was determined using Geltrex (Fig.2). A significant decrease in invasion was seen with both the MDA-MB-231 and MDA-MB-436 cells. There was also a 17% reduction in invasion with the BT-549 cells, but this did not reach statistical significance. There was no effect of knocking down NAT1 on chemotaxis, which was determined in the same experiments by omitting the Geltrex (data not shown) suggesting that loss of NAT1 did not alter cell migration.

### Loss of NAT1 alters N-cadherin/β-catenin and snail expression

**Figure 3:**
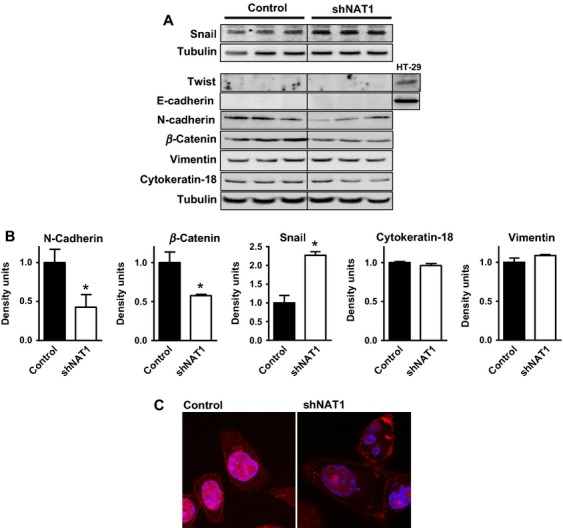
Effect of arylamine *N*-acetyltransferase I (NAT1) knockdown on gene expression in MDA-MB-231 cells. (A) Western blots demonstrate the expression of genes associated with epithelial to mesenchymal transition in breast cancer cells (*n* = 3). (B) Protein levels were quantified by densitometry and normalized to tubulin. Results are mean ± SEM, *n* = 3. Asterisk indicates *P *<* *0.05. (C) Staining of Control and NAT1 knockdown cells showed a reduction in the amount of *β*-catenin in the nucleus following NAT1 knockdown (red = *β*-catenin). The nuclei were stained with DAPI.

Invading breast cancer cells display a motile phenotype and a gene expression profile that includes extracellular proteases that degrade the surrounding tissue matrix 26. Invasion also has been associated with the transition from an epithelial phenotype to a more mesenchymal phenotype 27 and therapeutic strategies that induce the epithelial phenotype have been suggested as a means to prevent metastatic disease 28. Here, we investigated whether the reduced cell invasiveness following NAT1 knockdown in MDA-MB-231 cells was associated with mesenchymal–epithelial transition (MET) by examining several well-characterized MET markers (Fig.3A). There were no differences in cytokeratin-18 or vimentin expression (Fig.3B), and neither Twist nor E-cadherin was detected in control or shNAT1 cells (Fig.3A). By contrast, there was a significant increase in Snail expression while both N-cadherin and *β*-catenin were significantly decreased following NAT1 knockdown (Fig.3B). Both N-cadherin and *β*-catenin have been associated with breast cancer invasiveness 29–31 and are the targets for novel anticancer therapies 32,33. In MDA-MB-231 cells, *β*-catenin is mainly localized to the nucleus (Fig.3C). Upon NAT1 knockdown, there appeared to be a decrease in the nuclear content of this protein (Fig.3C).

### Loss of NAT1 decreases cell surface filopodia

**Figure 4:**
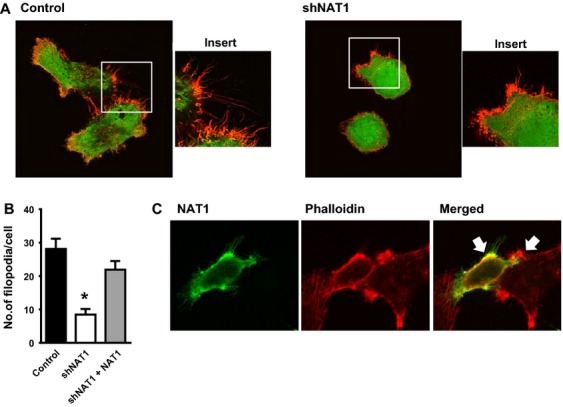
Loss of arylamine *N*-acetyltransferase I (NAT1) reduces the number of filopodia. (A) MDA-MB-231 cells stained with phalloidin show a decrease in filopodia protrusions following NAT1 knockdown (red = phalloidin, green = EGFP). (B) The number of filopodia was significantly reduced in the NAT1 knockdown cells compared to the control cells. Results are mean ± SEM, *n* = 9. Asterisk indicates *P *<* *0.05. (C) Reintroduction of NAT1 into the shNAT1 cells rescued the effect of NAT1 knockdown on filopodia and cell morphology (red = phalloidin, green = FLAG-NAT1).

Since a decrease in NAT1 activity induced a more rounded cell morphology and inhibited in vitro cell invasion, we investigated the cytoskeleton structure of the cells. NAT1 knockdown cells displayed significantly less filopodia compared to the control cells (Fig.4A). Quantification of filopodia showed that loss of NAT1 reduced the number of filopodia on each cell to less than 35% of controls (Fig.4B).

To determine if the loss of filopodia could be rescued by reintroducing NAT1, the NAT1 knockdown cells were transiently transfected with a vector-expressing FLAG-NAT1. Figure4C shows the presence of both transfected (green) and nontransfected cells. The filopodia associated with the lamellipodia (arrows) can be seen in those cells expressing NAT1 but to a lesser extent in neighboring cells devoid of the enzyme. Quantification showed that reintroduction of NAT1 rescued the NAT1 knockdown phenotype (Fig.4B). There was also a reversal of cell morphology to the more elongated shape of the control cells.

NAT1 expression showed a distinct subcellular distribution. The majority of the protein was localized to the cortical cytoskeleton and the lamellipodia F-actin (Fig.4C). There was also evidence for its presence in the filopodia protrusions.

### NAT1 knockdown inhibits in vivo metastasis

**Figure 5:**
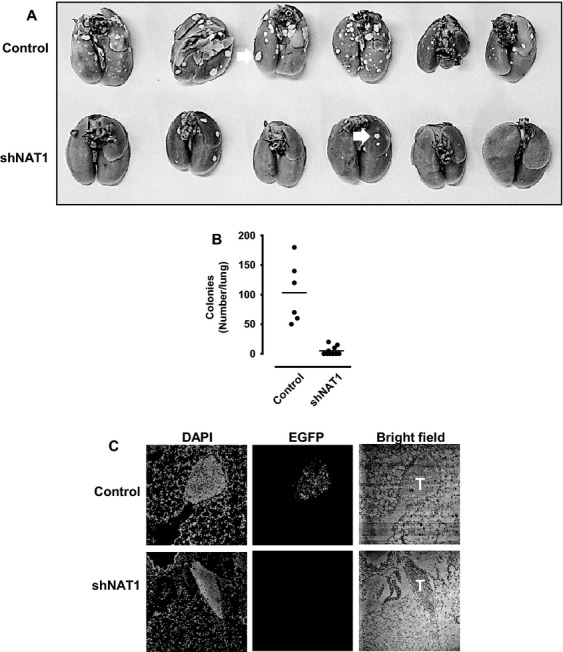
Loss of arylamine *N*-acetyltransferase I (NAT1) inhibits in vivo metastasis. (A) The control and NAT1 knockdown MDA-MB-231 cells were injected into the tail vein of nude mice and lungs were harvested 56 days postinjection. Macrometastases are shown by arrows. (B) Quantification shows that NAT1 knockdown significantly reduced the number of colonies formed in lung, *n* = 6–10. (C) Sectioned lung tissues viewed by confocal microscopy shows tumors (T) formed by control cells expressed EGFP while tumors from mice injected with shNAT1 cells have little or no EGFP expression. The nuclei were stained with DAPI.

To investigate the effects of NAT1 knockdown on metastatic potential in vivo, we employed a lung metastasis model in BALB/c nu/nu mice. Lentivirus-transduced control and shNAT1 MDA-MB-231 cells were injected intravenously and lung colonization was assessed 56 days later. In mice injected with control cells, numerous macroscopic colonies were seen (Fig.5A), ranging from 50 to 180 per lung. By contrast, the shNAT1-transduced cells resulted in significantly less colonies, ranging from 0 to 20 per lung (Fig.5B).

Since the lentivirus-transduced cells were tagged with EGFP, we examined EGFP expression in the metastatic colonies from control and shNAT1 mice (Fig.5C). Immunostaining showed the presence of EGFP in lung colonies from mice injected with the control cells. However, colonies in lung tissues sectioned from mice injected with shNAT1 cells expressed little or no EGFP. These results suggest that successful colony formation in animals injected with shNAT1 cells was due to the loss of shRNA expression in those cells.

## Discussion

NAT1 was first identified as a xenobiotic metabolizing enzyme that uses acetylcoenzyme A to transfer an acetyl group to arylamine and heterocyclic amine compounds. However, recently, its role in cell growth and survival has attracted growing interest 4. In the present study, we have shown that downregulation of NAT1 in breast cancer cells results in a marked change in morphology, a loss of surface filopodia and a decrease in invasive potential both in vitro and in vivo. The molecular mechanism(s) that underlie these effects are still not well understood. The only known endogenous substrate for NAT1 is the folate catabolite *p*-aminobenzoylglutamate 34. However, there are no known metabolic processes that link *p*-aminobenzoylglutamate to cancer cell proliferation or invasion. Moreover, recent studies in mice devoid of the NAT1 homolog (*Nat2*) showed no effect on tissue levels of *p*-aminobenzoylglutamate suggesting that acetylation of this substrate in vivo is not significant 35.

NAT1 knockdown resulted in a phenotypic change in all three cell lines studied. Most notable were the collapse of filopodia and a rearrangement of intracellular actin. Changes in invasion through Geltrex, however, were only observed for the MDA-MB-231 and MDA-MB-436 cells. While all three cell lines lack estrogen receptors, progesterone receptors, and HER2 (triple negative), they differ extensively in other aspects such as tissue origin and cancer type 36. In addition, only MDA-MB-231 and MDA-MB-436 cells carry a wild-type RB1 37 and both express low levels of *β*-catenin compared to BT-549 cells 38. All three lines differ considerably in their responses to chemotherapeutic agents 37. Interestingly, BT-549 have a much lower proliferative index compared to MDA-MB-231 and MDA-MB-436 cells 39, which has been associated with a decrease in invasiveness in breast cancer 40. The differences found in the current study may provide leads to the molecular events associated with NAT1 knockdown.

There is compelling evidence showing that EMT plays an important role in breast cancer invasion and metastasis, although cancer progression does not exclusively rely on this process 41. EMT is loosely defined as a change in cell morphology from a spindle shape to a more epithelial phenotype, repression of epithelial markers and gain of mesenchymal markers, and functional changes that enhance metastatic capacity 41. The change in cell morphology and reduced cell invasion following NAT1 knockdown in MDA-MB-231 cells led us to investigate a possible association between NAT1 and MET. However, we saw little or no change in expression of the common markers Twist, vimentin, and cytokeratin-18. Snail expression increased, while both N-cadherin and *β*-catenin were significantly reduced following NAT1 knockdown. Although detectable in MDA-MB-231 cells, N-cadherin is expressed at low levels and has been reported as absent by some investigators 42. Its expression has been associated with an increase in tumor cell invasion and metastasis 43. Similarly, *β*-catenin is involved in the upregulation of genes that favor invasion 44. Thus, the decrease in both proteins is consistent with the changes in invasiveness seen in the current study. Deciphering exactly how NAT1 may influence the expression of N-cadherin and/or *β*-catenin will be pivotal to understanding its effects on breast cancer cell morphology.

The mechanism by which NAT1 knockdown inhibits invasion and metastasis partly involves modulation of filopodia and is independent of cell migration. Filopodia are dynamic structures that can extend and retract 45. Their functions are not completely understood but they can act as sensory organelles that explore the external environment 45. Filopodial actin dynamics have been implicated in promoting cancer metastasis and invasion as proteins involved in filopodia formation have been shown to enhance cancer invasion and metastasis 46. Invading myofibroblasts express N-cadherin at the tips of their filopodia and inhibition by short interfering RNA reduces filopodia formation 47. Our data showed that NAT1 has a distinct subcellular localization with the highest levels associated with the lamellipodia (see Fig.4C). NAT1 was also seen in the filopodia extensions. This distribution of NAT1 may provide important clues to the function of NAT1 in cell growth, survival, and invasion. Acetylation has been associated with the formation of filopodia, stress fibers, and lamellipodia, which involves the small GTPases cdc42, RhoA, and Rac1 48. Each of these is regulated by the inhibitor RhoGDI and acetylation of RhoGDI prevents its interactions with the small GTPases. Deacetylation of RhoGDI leads to a decrease in filopodia formation 49. Further work is required to determine if there is a link between NAT1 activity and small GTPase function.

In summary, the present study demonstrates a role for NAT1 in breast cancer metastasis that may be independent to its role in arylamine metabolism. This effect was not seen in all triple-negative breast cancers. NAT1 inhibition does not appear to be detrimental to normal cells because mice lacking the murine homolog of NAT1 are mostly normal in appearance, behavior, mortality rate, and reproductive capacity 50. This makes NAT1 an attractive drug target in cancer therapeutics.
